# Investigating the use of low and high-density polyethylene blends with waste material from three-layer factory tube for the third layer of shock tubes

**DOI:** 10.3389/fchem.2025.1545984

**Published:** 2025-03-19

**Authors:** Ali Khalili Gashtroudkhani, Mohammad Dahmardeh Ghaleno, Saeed Soltan Abadi, Maryam Pouyani

**Affiliations:** ^1^ Faculty of Polymer Processing, Iran Polymer and Petrochemical Institute, Tehran, Iran; ^2^ Department of Wood and Paper Sciences and Technology, University of Zabol, Zabol, Iran; ^3^ Faculty of Management and Industrial Engineering, Malek Ashtar University of Technology, Tehran, Iran; ^4^ Department of Wood and Paper Sciences and Technology, Gorgan University of Agriculture Sciences and Natural Resource, Gorgan, Iran

**Keywords:** blasting systems, shock tube, polymer compound, mechanical properties, low density

## Abstract

Polymeric shock tubes are now widely used in explosives systems for drilling and mining operations. Most shock tubes on the market consist of three layers of polymer, the first layer being Surlyn 8940 copolymer, the second layer Nucrel 31001 and the outer layer Borostar ME 6053 medium density polyethylene. Surlyn and Nucrel are usually sourced from DuPont, polyethylene from Charlotte Boralis. the main goal in this research is reducing the price of final shock tube and reuse the waste tube of plant (rejected shock tube) with improving the properties of product. For reaching to this goal, using polyethylene blend with available raw materials in the country and mixing them with rework from the shock tube production plant. For this purpose, different proportions of low- and high-Density polyethylene are blend using a twin-screw extruder and finally mixed with some of the factory’s polymer rework. In the first phase, the low-density polyethylene LDPE 020, the high-density polyethylene HDPE HI 0500 and the filler calcium carbonate were blend in a twin-screw extruder and compounded with different percentages of 20/75/5, 30/65/5, 40/55/5 and 47/47/6 percent respectively. In the second phase, the resulting blend was mixed physically with 5, 10 and 15 percent three-layer tube rework (which was crushed with a crusher or pelletizer). The results showed that the 47/47/6 percent mixture had the best composition in terms of the production process, the properties of blend in terms of tensile strength (17/3 MPa), elongation percentage (458%) was suitable. In order to reduce the waste and cost of the product, the best processing results, product properties and costs are obtained when the above composition is mixed with crushed shock tube rework in a ratio of 90/10 (blend/rework). Tensile strength at break was 20/01 MPa and elongation at break was 478%. After evaluating the raw materials and accepting the results, the polymer blends were used on an industrial scale to produce shock tubes. The performance of the resulting shock tubes was then compared using various tests, including mechanical tests, oil penetration resistance, thermal shrinkage (in 60°C: upper 7% and in 80°C: upper 9%), burst strength, thermal aging (before aging:170 N, after aging: N_5_, N_6_, N_7_, N_8_: upper 170 N), and explosion velocity (upper 1890 m/s). The results showed that by using the polymer blend with rework, the mechanical properties of the shock tubes produced met the standard (tensile strength of more than 170 N/m^2^ and elongation percentage of more than 220). The results of the oil penetration resistance (45–50 h), burst strength and aging tests also showed that all shock tubes manufactured with the new third layer had acceptable properties and were on the same level as shock tubes made of Boralis polyethylene.

## Introduction

**FIGURE 1:**
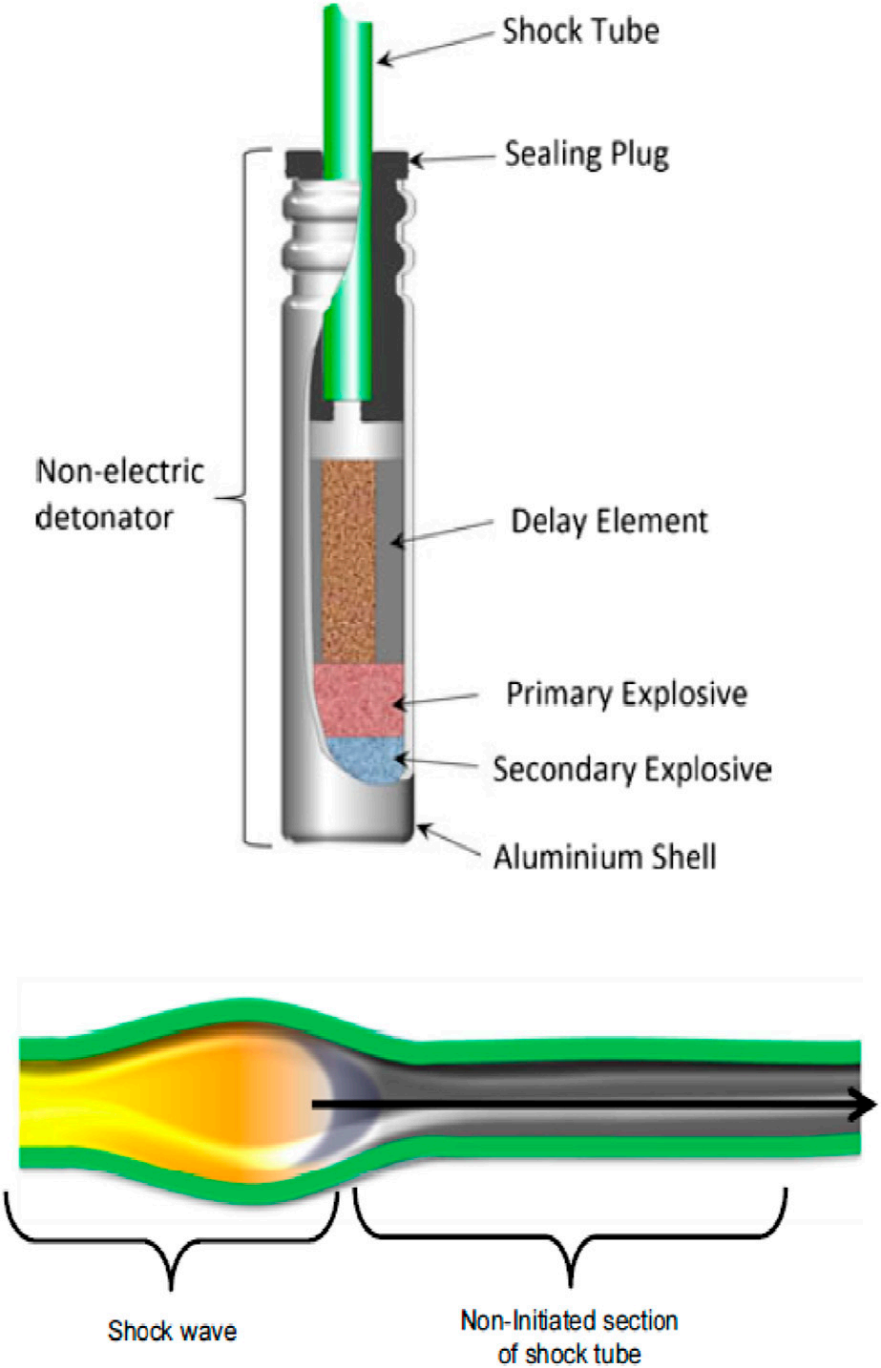
Above side of the shock tube connected to a non-electric explosive detonator (Yakan-A-Nwai and Mukhopadhyay, 2013a) and below side of the shock wave transmission inside the tube by the combustion of explosive powder inside it (Balan and Raj, 2023; Yakan-a-Nwai and Mukhopadhyay, 2013b).

One of the most important developments in the 1960s in the mining, construction and drilling sectors was the industrial development of the shock tube detonator (Yakan-A-Nwai and Mukhopadhyay, 2013a; AyalaCarcedo, 2017; Persson et al., 2018; Himanshu, 2024). In general, explosive systems (blasting systems) used in mining and drilling operations consist of three parts: the detonator, the explosive charge and the energetic charge (Balan and Raj, 2023; Li et al., 2024). The function of the detonator is to emit a signal or wave that activates and sets off the explosive charge. The explosive charge receives the signal and, by amplifying it, causes the energetic charge to explode (Yakan-A-Nwai and Mukhopadhyay, 2013a; AyalaCarcedo, 2017; Himanshu, 2024; Dick et al., 1982). Shock tubes are a type of initiator system. These devices consist of a hollow plastic tube containing explosive powder (see in Figure 1) (Yakan-A-Nwai and Mukhopadhyay, 2013a; Fordham, 2013; Choudhary, 2024; Meyer et al., 2016).

Polymer shock tubes can be divided into three categories depending on the number of layers: single-layer, double-layer and triple-layer (Salehi et al., 2021; Jia et al., 2024; Abedi et al., 2018). Gladden et al. developed a triple-layer shock tube in 2002 (Yakan-a-Nwai and Mukhopadhyay, 2013b; Goncharov, 2023). Gladden et al. used ethylene-Surlyn ionomers for the first layer, which should have good adhesion to explosive powders. For the outer layer, they proposed nylon 6, LDPE and MDPE (Yakan-a-Nwai and Mukhopadhyay, 2013b; Goncharov, 2023). For the middle layer, they also selected polymers with good adhesion to the other two layers (Yakan-a-Nwai and Mukhopadhyay, 2013b; Goncharov, 2023).

In 2013, Christian Yakan et al. used the following compositions for the second layer of a two-layer shock tube (Yakan-A-Nwai and Mukhopadhyay, 2013a; Yakan-a-Nwai and Mukhopadhyay, 2013b):

HDPE/HDPE g MAH: 30/70

HDPE/IONOMER: 30/70

HDPE/ EMA: 30/70

LLDPE/ IONOMER: 30/70

After fabricating the two-layer shock tube, they concluded that the first three compounds, most of which are made of HDPE, have higher burst strength than the last compound, which is made of LLDPE, due to less branching and greater crystallinity.

The sleep time of the HDPE/HDPE g MAH blend was also higher than that of the other blends, with maleic anhydride groups increasing the penetration resistance and thermal resistance of the polymer. High thermal resistance reduces heat transfer from hot oil to the inner layer of the shock tube, and hot oil takes longer to transfer to the inner layer. The blend made from high density polyethylene and ionomer has moderate penetration resistance due to the complex molecular structure of the ionomer, which makes it difficult for organic and inorganic solvents to penetrate it. They also found that the blend of high-density polyethylene and ethylene methacrylate has low penetration resistance and the blend of linear low-density polyethylene has the lowest oil penetration resistance, which is due to the presence of civet branching and consequently more pores in linear low-density polyethylene that allow solvent penetration.

They also found that the breaking strength of the blend of high-performance polyethylene and ethylene methacrylate was higher than that of the others. This is due to the high adhesion of these two compounds to each other. Today, conventional and commercially available shock tubes are available in single-layer, double-layer and triple-layer types. However, high-performance shock tubes usually consist of three polymer layers. The performance characteristics of a triple-layer shock tube are listed in Table 1. The inside of such tubes is coated with a mixture of HMX and aluminum as an explosive powder (Yakan-a-Nwai and Mukhopadhyay, 2013b; Manner et al., 2012). Different types of Surlyn are used to produce the inner layer of this type of shock tube. When selecting the polymer used for the outer layer, polyolefins are usually preferred due to their low price and easy and cheap processability. In this case, the middle layer must be able to create good adhesion between a polar polymer such as Surlyn and a non-polar polymer such as the polyolefins. NUCREL is an ethylene copolymer that is copolymerized with different proportions of acrylic acid or methacrylic acid, depending on the type and grade. In most shock tubes, this copolymer is used as the raw material for the middle layer. However, the price of this copolymer is higher than that of conventional polyolefins, so replacing it with a cheaper polymer with the same performance characteristics can reduce production costs.

**TABLE 1:** Summary of average properties of commercial high-performance three-layered shock tubes.

Properties	Approximate range
Breaking strength (rate of extension: 70 mm/min)	160 N–200 N
Elongation at break (rate of extension: 70 mm/min)	200%–300%
Linear thermal shrinkage (After 1 h in an oven @ 80 ℃ )	6%–10%
Oil penetration (50 ℃ in Paraffin)	45–50 h
Burst strength (After 20 min in an oven @ 80 ℃ )	Normally 2–5 bursts per 5 m of tubing
Velocity of detonation (over a length of 60 cm)	1800–2200 m/s

Polyolefins are among the most widely used thermoplastics, which are widely used in the packaging, wire and cable, automotive, electronics and other industries due to their low price, good low-temperature processability and easy availability (Parthasarathy et al., 2013; Rajeshwari and Dey, 2014; Graziano et al., 2020; Rajeshwari et al., 2019; Favakeh et al., 2020).

However, some properties of these materials prevent their wider use. In the polymer industry, a material with suitable properties can be obtained by blending and compounding two or more polymers. The properties of the blend resulting from the properties of the individual blend phases in the pure and single state are thus better. Due to the good compatibility of the polyolefin family, the alloying of different polyethylene types with each other has been carried out for years on an industrial and laboratory scale (Rosales et al., 2020; Su et al., 2010; Klimovica et al., 2020; Zhou et al., 2020; Leng et al., 2020). In plants for the production of shock tubes, a large amount of rework is usually generated at the beginning of the production process. The addition of this rework, which contains Surlyn ionomer, interlayer adhesive and three-layer polyethylene, to the polyethylene mixture can increase the modulus and tensile strength of the polyethylene (Rosales et al., 2020; Su et al., 2010).

## Experimental

### Materials

Ionomer Surlyn 8940 with a density of 0.95 g/cm^3^ and a melt flow index of 2.8 dg/min containing sodium ions and methacrylic acid groups, as well as Nucrel 31,001 with a density of 0.940 g/cm^3^ and a melt flow index of 1.3 dg/min containing 9.5 wt% acrylic acid groups, were obtained from DuPont, United States. Medium density polyethylene with trade names HTA002, EXCEES 1327CA, Borstar ME6053 was obtained from Boralis.

### HTA002

It is a type of high density, medium molecular weight polyethylene that is easy to process. This polymer is normally used in coextrusion processes with other polyolefins or alone. Its physical properties are shown in Table 2. In the structure of the shock tube, it is used in the production of the material composition of the outer layer of the shock tube. It accounts for 30% of the weight of this material.

**TABLE 2:** Physical specifications of HTA002.

Property	Value	Description
Density	0.95	
Melt flow rate (MFR)	0.69 g/10 min	
High load melting coefficient (LMI)	16 g/10 min	
Softening Point	126 ℃	
Maximum process temperature	285 ℃	Recommended extruder temperature: First zone 135 ℃ Second zone 169 ℃ Third zone 185 ℃ Fourth zone to die 185 ℃
Mechanical properties of the film	-	Tensile Strength at Yield, MD: 29 MPa Tensile Strength at Break, MD: 60 MPa Module 960 MPa

### Exceed 1327CA

This polymer is a hexane-ethylene copolymer produced with a metallocene catalyst. It has a high modulus and at the same time a high toughness and is therefore ideal for the production of multilayer films. The interesting thing about this material is that no data sheet has been published for it.

### Borstar ME6053

This material is a type of medium-density polyethylene that is characterized by its resistance to ultraviolet radiation and its good colourability. This material is manufactured using Borstar’s special technology, which gives it the ability to facilitate processability and molding. The shrinkage of this part during molding, common in polymers, is reduced to a minimum while maintaining mechanical strength. It contains ultraviolet-resistant fillers. Typically, one of Exceed 1327CA and Borstar ME6053 selectively makes up 70% by weight of the outer layer of the shock tube (As seen in Table 3).

**TABLE 3:** Physical specifications of Borstar ME6053.

Property	Typical value	Test method
Density	936 kg/m^3^	ISO 1183-1 Method A
Melt Flow Rate (190 ℃ /2.16 kg)	0.7 g/10 min	ISO 1133-1 Method A
Melt Flow Rate (190 ℃ /5.0 kg)	3 g/10 min	ISO 1133-1 Method A
Flexural Modulus	600 Mpa	ISO 178
Tensile Strain at Break (50 mm/min)	800%	ISO 527-2
Tensile Strength (50 mm/min)	32 Mpa	ISO 527-2
Brittleness Temperature	< −76 ℃	ASTM D 746
Environmental Stress Crack Resistance (50 ℃ , lgepal 10%, F0)^a^	>5,000 h	IEC 60811-406
Hardness, Shore D (1 s)	54	ISO 868
Pressure Test at High Temperature (115 ℃ , 6 h)	<10%	IEC 60811-508

^a^ No crack.

Typically, one of the two materials Exceed 1327CA and Borstar ME6053 selectively makes up 70% by weight of the outer layer of the shock tube.

### Sarmatene

This material is added as a pigment to the polymer mixture mentioned in the previous step. It is a colored low-density polyethylene that is added to the mixture of HTA0200 and Borstar ME6053 by applying color. Its color depends on the user’s choice and varies. Its physical properties are listed in Table 4.

**TABLE 4:** Physical properties of the pigment.

Property	Value
Density	0.91 g/cm^3^
Melt flow rate (MFR)	7 g/10 min

In this research work, based on the study of the properties of different types of polyethylene, two types of low- and high-density polyethylene obtained from Bandar Imam Petrochemical Company were used. The properties of the light polyethylene LDPE020 and heavy polyethylene HDPE HI 0500 used in this study are shown in Tables 5, 6:

**TABLE 5:** The characteristics of low density polyethylene LDPE020 and high density polyethylene HDPE HI 0500.

Light polyethylene LDPE020			
Property	Unit	Value	Test Method
MFI (190 ℃ /2.16 kg)	gr/10 min	2	ASTM D 1238
Density	gr/ml	0.920	^a^TSTM 209 B
Softening Point	℃	94	ASTM D 1525
Haze	%	15 max	ASTM D 1003
Gloss @ 60	Gu	60 min	ASTM D 523
Elongation @ break (MD)	%	330 min	ASTM D 882
Elongation @ break (MD)	%	600 min	ASTM D 882
Tensile @ break (MD)	kg/cm	160 min	ASTM D 882
Dart Impact	Gr	100 min	ASTM D 1709

^a^ TSTM = toyo sods standard test method.

**TABLE 6:** The characteristics of high density of polyethylene HDPE HI 0500.

Heavy polyethylene HDPE HI 0500			
Property	Unit	Value	Test Method
Mass Density (23 ℃ )	g/cm^3^	0.963–0.967	ASTM D 1505
Melt Flow Rate (190 ℃ /2.16 kg)	g/10 min	4–6	ASTM D 1238
Ash Content	wt%	0.06 Max	ASTM D 1063
Volatile Matter	wt%	0.05 Max	ASTM D 1960
Tensile Strength@ break	g/cm^2^	170 Min	ASTM D 638
Elongation @ break	%	300 Min	ASTM D 638
Melting Point	℃	130	ASTM D 2117
Vicat Softening Point	℃	124	ASTM D 1525
ESCR	hr	4	ASTM D 1693

### Middle layer

The middle layer is generally an adhesive used to bond the outer and inner layers of the shock tube. Its trade name is Nucrel 31,001 and this material is a type of ethylene-acrylic copolymer containing 9.5% acrylic acid by weight. Its main application is injection molding and coextrusion as an adhesive. Its physical properties are listed in Table 7.

**TABLE 7:** Physical specifications of Nucrel 31001.

Property	Value	Description
Density	0.94	
Melt flow rate (MFR)	1.34 g/10 min	
Melting Point	99 ℃	
Softening Point	79 ℃	
Maximum process temperature	285 ℃	Recommended extruder temperature: First zone 135 ℃ Second zone 169 ℃ Third zone 185 ℃ Fourth zone to die 185 ℃

In this study, the Nucrel copolymer was replaced by a blend of polyethylene grafted with maleic anhydride and polypropylene and used as a second layer. Its properties are given in Table 8.

**TABLE 8:** Physical specifications of polyethylene grafted with maleic anhydride (PEgMA).

Property	Value	Description
Density	0.945	
Melt flow rate (MFR)	1.8 g/10 min	
Melting Point	105 ℃	
Softening Point	75 ℃	
Maximum process temperature	140–190 ℃	Recommended extruder temperature First zone 140 ℃ Second zone 175 ℃ Third zone 185 ℃ Fourth zone to die 190 ℃

### Inner layer

This layer consists of two parts: Polymer and explosive. The polymer part is the main carrier and the aluminum powder and the HMX explosive are the materials that come into contact with the tube. Surlyn 8940 is the actual carrier of the explosive in the shock tube. The trade name of the material is Surlyn 8940, which is a thermoplastic sodium ionomer plastic produced by copolymerization of methacrylic acid in the presence of ethylene so that it contains 14.5%–15.5% methacrylic acid by weight and its acid groups are neutralized by sodium ions. In fact, an acidic salt is formed at the end. Table 9 shows the physical and process properties for identification. The main application of this material is in extrusion molding.

**TABLE 9:** Physical specifications of Surlyn 8,940.

Property	Value
Density	0.95
Melt flow rate (MFR)	2.80 g/10 min
Melting Point	94 ℃
Softening Point	63 ℃

### Explosive

The inner wall of the inner layer of the shock tube is coated with explosive powder. This powder mixture is in micronized form. The powder consists of 92% HMX and 8% aluminum powder.

### Preparation of samples

With the help of a ZSK twin-screw extruder (ZSK MC^18^ D_0_/D_i_:1/55^)^ from Iran Polymer and Petrochemical Institute, low density polyethylene LDPE 020, high density polyethylene HDPE HI0500 and filler were blend as follows:

• 20/75/5%
• 30/65/5%
• 40/55/5%
• 47/47/6%

**FIGURE 2:**
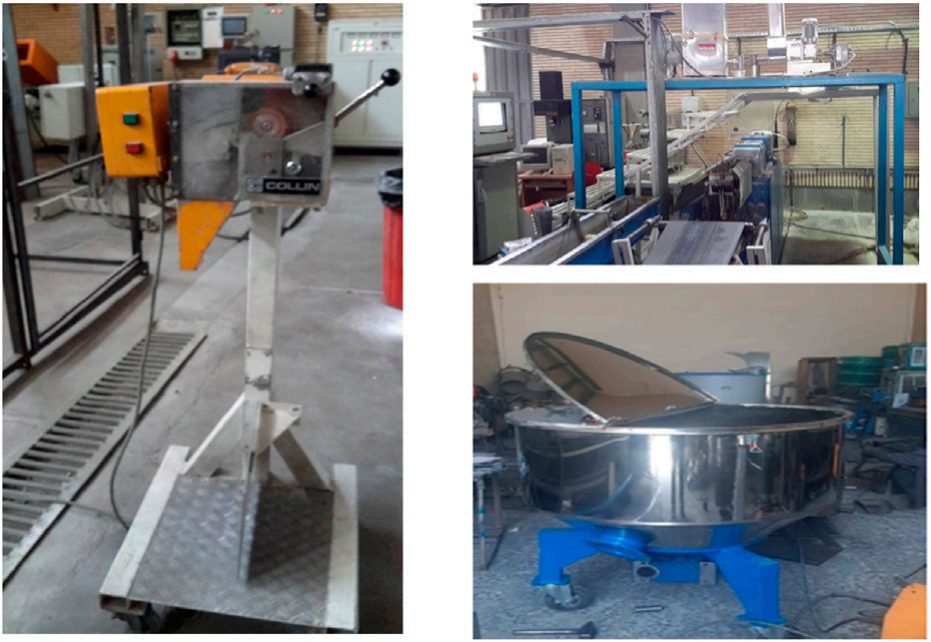
ZSK twin-screw extruder (Top), Mixer (Bottom) of polymers and Shock Tube three-layer tube rework crushing machine (Left).

The three-layer shock tube rework produced in the factory was broken down into 1*3 mm particles using a crushing machine (Pelletizing System COLLIN SP2) (As seen in Figure 2). In the next step, the best compound in terms of process, properties and final price was mixed with shredded three-layer tube rework at 5, 10 and 15 percent respectively using a mixer (Horizontal behsaz polymer 100 Kg) (As seen in Table 10). The specific condition for mixing the best blend with rework:

**TABLE 10:** Composition percentage of components used to prepare the blend.

Sample	LDPE	HDPE	Filler	Rework
P1	20	75	5	-
P2	30	65	5	-
P3	40	55	5	-
P4	47	47	6	-
P5	44.65	44.65	5.70	5
P6	42.3	42.3	5.4	10
P7	39.95	39.95	5.1	15
P8	-	-	-	-

RPM: 50

Temperature: 25°C

Time: 1 h.

Stages of adding rework to blend: 3 stage.

The polyethylene control sample was Borostar medium-density polyethylene labeled P8.

After preparing the polymer blends, 100 m shock tubes of each of the formulations were prepared according to the method described by Yakan and Mukhopadhyay (Yakan-A-Nwai and Mukhopadhyay, 2013a). The shock tubes were named according to Table 11.

**TABLE 11:** Nomenclature of manufactured shock tubes.

Shock tubes	First layer	Second layer	Third layer
Shock Tube N1	Surlyn 8940	PEGMA	P1
Shock Tube N2	Surlyn 8940	PEGMA	P2
Shock Tube N3	Surlyn 8940	PEGMA	P3
Shock Tube N4	Surlyn 8940	PEGMA	P4
Sho ck Tube N5	Surlyn 8940	PEGMA	P5
Shock Tube N6	Surlyn 8940	PEGMA	P6
Shock Tube N7	Surlyn 8940	PEGMA	P7
Shock Tube N8	Surlyn 8940	PEGMA	P8

## Methods

### Mechanical properties

First, dumbbell-shaped samples of Borstar medium density polyethylene (P8) and samples P1 to P7 were prepared using a injection molding machine available in Iran Polymer and Petrochemical Research Institute according to ASTM 823 standard. Then, 5 dumbbells from each of the samples were subjected to mechanical tensile testing using a SANTAM STM-20 tensile testing machine at a speed of 100 mm/min. In addition, in order to examine the mechanical properties of the manufactured shock tubes and after heat aging, 30 cm long samples were cut from each of the shock tube tubes N1 to N8 and subjected to mechanical tensile testing at a speed of 100 mm/min.

### Linear thermal shrinkage test

This test was carried out at two temperatures of 60 ℃ and 80 ℃ . In this way, 10 shock tubes with a length of 30 cm were cut from each sample. Then their two ends were welded with an ultrasonic device to completely seal the two ends of the tubes. A distance of 20 cm was then marked on all samples. In the next step, the samples were placed in a heating oven for 1 hour, once for the 60 ℃ test and once for the 80 ℃ test. After the samples were removed, the marked distances were measured again. Due to thermal shrinkage, this distance should be less than 20 cm after heating the shock tube in the oven. Using Equation 1 and multiplying the result by 100, the percentage of linear heat shrinkage of the samples was determined. In this relationship, L_0_ is the initial distance between the marks, which was assumed to be 20 cm, and L is the secondary distance between the marks that occurs after the tubes leave the oven.

$$\text{Linear shrinkage}=\frac{\left(L0-L\right)}{\left(L0\right)}$$ (1)

### Thermal aging test

As explained in the “Mechanical properties” section, for this test a certain number of samples from each of the shock tubes were cut to a length of 30 cm and placed in an oven at 50 ℃ for 8 h. They were then subjected to a tensile test. In addition, a number of samples from each of the shock tubes were detonated after leaving the furnace to determine the effects of thermal aging on the explosive properties of the samples.

### Burst strength

**FIGURE 3:**
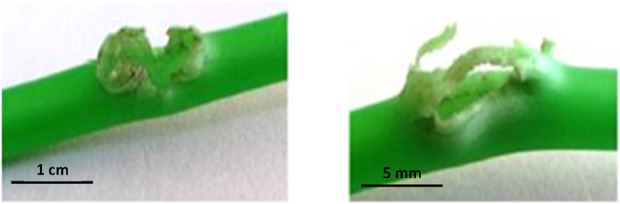
Image of two tubes that burst during the burst strength test (Yakan-A-Nwai and Mukhopadhyay, 2013a; Yakan-a-Nwai and Mukhopadhyay, 2013b).

When the shock tube is activated, the explosive powder in the inner layer explodes and transmit to the end of the tube until the wave resulting from the explosion of the explosive powder reaches the primer and triggers it. For the shock tube to function properly, it is necessary that the shock wave is only transmitted in the longitudinal direction. Therefore, the tube wall must resist the shock wave entering in the radial direction. This resistance is referred to as bursting strength. To test the bursting strength, three samples of 5 m length were cut from each type of tube. The samples were placed in an oven at 50 ℃ for 20 min. Then they were removed from the oven and exploded with the help of an exploder, and the bursting or non-bursting of the tubes in the radial direction was visually inspected. According to Yakan and Mukhopadhyay (Yakan-A-Nwai and Mukhopadhyay, 2013a; Yakan-a-Nwai and Mukhopadhyay, 2013b). If the shock tubes have a low burst strength, they burst, as shown in Figure 3. The lower the number of bursting points in the tube wall, the more burst-resistant the shock tube is.

### Oil penetration resistance test

To carry out this test, 300 mL of liquid paraffin oil was poured into a beaker with a volume of 750 mL. Then a certain number of 30 cm long shock tubes, both ends of which were sealed with an ultrasonic device, were placed in a U-shape in the beaker with oil. The middle 10 cm of the tubes came into contact with the paraffin oil. So that the middle 10 cm of the tubes come into contact with the paraffin oil. The beaker was placed in a water bath and the temperature of the water in it was adjusted so that the temperature of the oil bath in the beaker remained constant at 50 ℃ . The nature of the test is that the samples are removed from the oil bath at specific time intervals and tested for their explosiveness. This continues until the sample taken is no longer explosive. This means that the oil has penetrated the inner layer of the tube and prevented the tube from functioning properly.

## Results and discussion

### Mechanical properties

**FIGURE 4:**
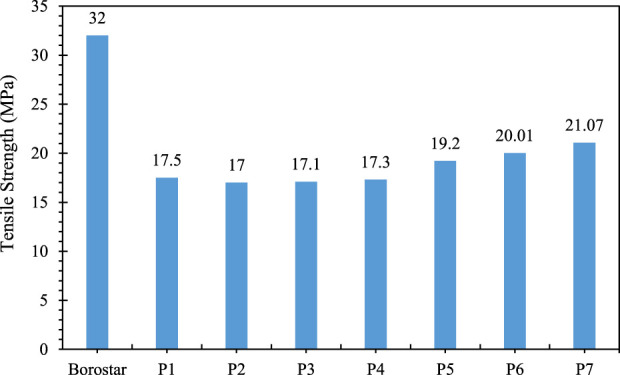
Tensile strength of Borostar polyethylene, blend of polyethylene compounds and shock tube rework.

**FIGURE 5:**
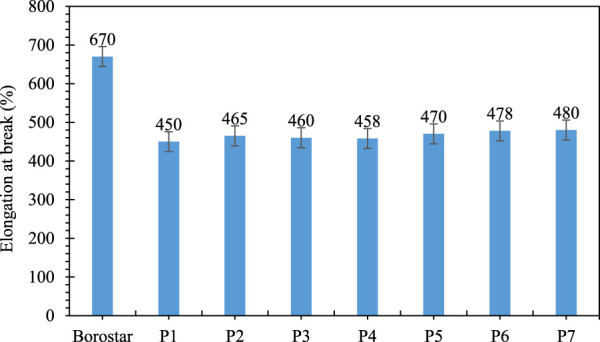
Percentage elongation of Borostar polyethylene, blend of polyethylene compounds and shock tube rework.

As can be seen in Figures 4, 5, when low- and high-density polyethylene are blend in different proportions, there is no significant change in the tensile strength and elongation of these two types of polyethylene as a percentage (Table 12). However, when three-layer shock tube rework is added, there is significant change in the tensile strength and elongation of between without and with rework and the tensile strength of the blend increases by two to four units, which is due to the presence of Surlyn ionomer in the rework, which has high mechanical strength. The elongation percentage of the compound also increases by 10–16 unit, which is due to the presence of Surlyn ionomer in the rework, which has a high elongation percentage.

**TABLE 12:** Result of analysis of variance effect of blending in tensile properties.

First column	Sum of squares	D.F	Mean squares	F value	Sig
Within group	0/442	3	0/147	0/037	0/990
Between group	32/000	8	4/000		
total	32/443	11			

The result has been showed that there is not no significant difference between various blending in tensile properties of product (Table 13).

**TABLE 13:** Result of analysis of variance effect of blending without and with rework in tensile properties.

First column	Sum of squares	D.F	Mean squares	F value	Sig
Within group	48/031	6	8/006	2/001	0/134
Between group	56/000	14	4/000		
total	104/031	20			

The result has been showed that there is not significant difference between various blending and using of rework in tensile properties of product.

### Linear thermal shrinkage

**FIGURE 6:**
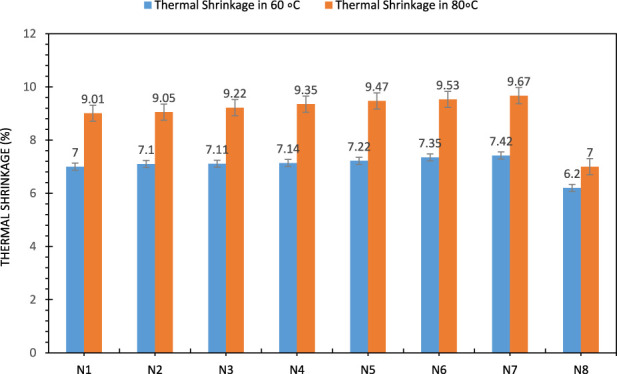
Percentage of thermal shrinkage of shock tubes at temperatures of 60 ℃ and 80 ℃ .

Polymer fibers and films that are stretched during the manufacturing process shrink when used at high temperatures (Leng et al., 2020; Gupta et al., 1994). Shock tubes are also stretched at various stages of the manufacturing process. In addition, the polymer chains are stretched and aligned in the extruder and as they exit the die. This alignment can improve the mechanical properties, but also leads to thermal shrinkage of the polymer. The results of the linear heat shrinkage test in Figure 6 show that the linear heat shrinkage of N1 – N4 is lower than that of the other shock tubes at both temperatures. At 60 ℃ , however, this difference is smaller. The higher thermal shrinkage at 80 ℃ compared to 60 ℃ is due to the polymer chains having more energy at higher temperatures to change their conformation and release some of the conformations formed during processing. Therefore, the polymer chains exhibit greater thermal shrinkage at higher temperatures at the same time.

However, samples N5, N6 and N7 exhibit greater thermal shrinkage than the other shock tubes. In ordered polymers, such as polymer fibers, both the amorphous and crystalline regions become ordered (Trznadel and Kryszewski, 1992). As the temperature increases, the polymer chains have the opportunity to escape from some of the induced arrangements. It is in the nature of things that the chains in the crystalline regions need higher temperatures or more time to escape from the arrangements. A polymer with lower crystallinity therefore shows greater thermal shrinkage at the same time and temperature. From this it can be concluded that the higher the crystal content in a particular polymer, the less linear thermal shrinkage this sample will exhibit under the same conditions as the same polymer with lower crystallinity. In addition to polyethylene, samples N5, N6 and N7 also contain a 5 to 15 ionomer compound of Surlyn and polyethylene grafted with maleic anhydride. The grafting of polar groups onto a semi-crystalline polymer chain such as polyethylene leads to a decrease in the sequence of chains that have to be joined together to form a crystal, and thus also in the degree of crystallinity of the polymer. Since the shock tubes produced in samples N5, N6 and N7 contain polar compounds and have a lower crystallinity, this conclusion can probably be used as a reason for the higher heat shrinkage of these three compounds compared to other shock tubes. Overall, the percentage of heat shrinkage of the tubes was within the normal range, i.e., less than 10.

### Mechanical properties of shock tubes before and after thermal aging

**FIGURE 7:**
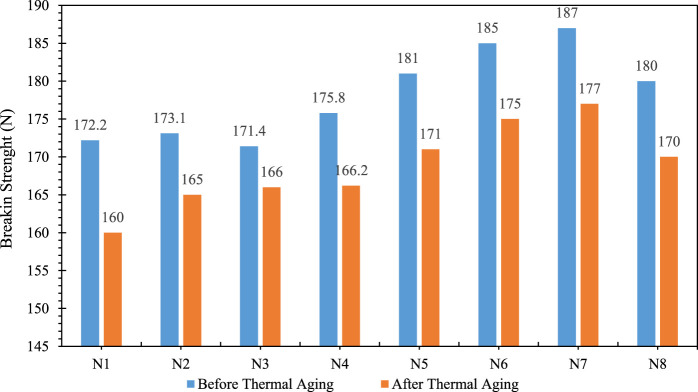
Force required to break shock transmission tubes, before and after thermal aging.

**FIGURE 8:**
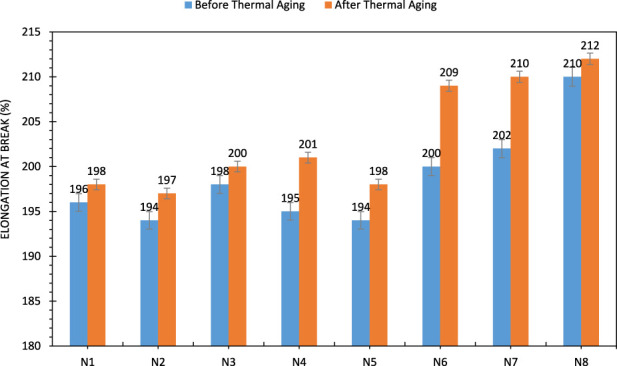
Breaking strain of shock tubes, before and after heat aging.

The tensile strength range of high-performance shock tubes is between 170–200 N (Yakan-a-Nwai and Mukhopadhyay, 2013b). The results of the tensile tests show that all shock tubes developed in this work have a tensile strength of more than 170 N. However, the shock tubes N5, N6 and N7 had higher tensile strengths than the other shock tubes. However, this value was not significantly different from that of the other samples, and it can be said that the shock tubes N1 to N4 have mechanical properties and breaking strength of 170–175 N, but when crushed rework is added to the third layer, the breaking strength of the tube increases to over 180 N. As mentioned above, the number of polar groups in Surlyn and PEGMA increases the adhesion between the second and third layers. Despite the higher tensile strength of the polyethylene blend, shock tube N8 has a lower tensile strength than N5, N6 and N7. Figures 7, 8 show that these results also apply to the tensile strength of shock tube. It can therefore be concluded that for the materials that make up the third layer of shock tube, a higher adhesive strength is likely to have a greater effect on improving the mechanical properties of shock tube than better mechanical properties. All shock tubes exploded after leaving the furnace with the help of an electric shocker. This indicates that thermal aging did not affect the explosive properties of the shock tubes. Thermal aging of blend is molecular deterioration as outcome of overheating due to the presence of tertiary hydrogen atoms in the polymer chan. The elevated temperature causes chain scission of the long chain backbone of polymer that react with one another. The mechanical properties of the shock tubes after thermal aging also showed a similar trend to the mechanical properties before thermal aging. However, after thermal aging, the tensile strength of all shock tubes decreased compared to before thermal aging. In addition, the tensile strength of the shock tubes increased.

The occurrence of linear heat shrinkage can affect the mechanical properties of shock tubes. Heat aging creates exactly the same conditions as linear heat shrinkage. The difference is that the samples in this test were stored in an oven at 50 ℃ for 8 h. The polymer materials that make up the shock tubes therefore had more time to relax and change their conformation. When the right conditions are created for a polymer to relax, the chains can be freed from some of the alignments created during the process. The reduction in alignment can lead to a decrease in the tensile strength of the polymer. On the other hand, the distance between the two ends of the chains is reduced as they tend to take on a spiral shape. This reduction in length results in the samples having a higher tensile strength in a tensile test.

### Bursting strength

In the case of multi-layer tubes, all layers must have good burst strength. The results of this test for all shock tubes showed that no bursting was detected in the samples, which means that all shock tubes have good burst strength.

### Oil penetration resistance test

The results of the oil penetration resistance test showed that all shock tubes could properly explode after 50 h in a paraffin oil bath at 50 ℃ and performed equally well. The correct performance of the shock tubes after this period against hydrocarbon oil at 50 ℃ means that the oil was not able to penetrate the inner layer of the shock tubes which is in contact with the explosive powder. All eight types of shock tube differed only in the material of the third layer. In these tubes, the second and first layers are polar in nature and have a higher oil resistance than MDPE. Therefore, even if oil penetrates the third layer, there are two additional polymer layers that prevent the penetration of oil molecules. According to Yakan and Mukhopadhyay (Yakan-a-Nwai and Mukhopadhyay, 2013b), high performance shock tubes should resist oil penetration for 45–50 h. Accordingly, tubes N1–N8 have sufficient resistance to oil penetration. This test lasted no longer than 50 h. Compared to most of the double-layered shock tubes produced in the research work of Yakan and Mukhopadhyay (Yakan-A-Nwai and Mukhopadhyay, 2013a), the shock tubes produced in this work have higher oil penetration resistance. This indicates that the presence of an additional layer in the three-layer tubes can effectively influence the oil penetration resistance of the shock tubes.

### Explosion velocity

**FIGURE 9:**
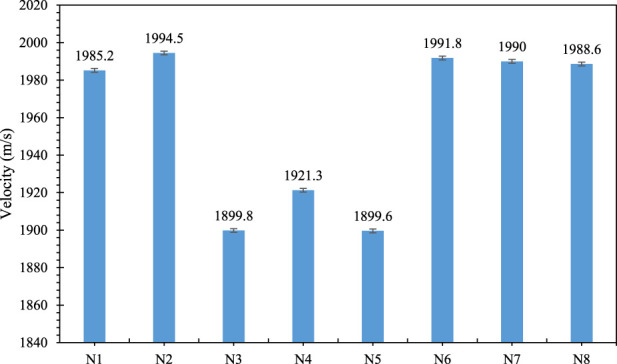
Shock tube explosion velocity in different samples.

Figure 9 shows the explosion velocity of shock tubes made with the third layer with different compositions. As can be seen, the explosion velocity of all tubes is higher than 1800 m/s. It can be concluded that the third layer has no effect on the explosion velocity of the shock tube and only affects the mechanical properties and resistance to solvent penetration.

## Conclusion

1. The results of the mechanical tests show that the addition of three-layer shock tube rework to the LDPE and HDPE blend increases the mechanical tensile strength and the percentage elongation of the finished tube. However, the best percentage of rework is 10%. This is because when the second and third layers are added to the Surlyn tube in the coextruder section, 15% of the coextruder outlet is extruded as filamentous melt, causing the tube to crack during production and stopping the production line, which in turn increases production rework.
2. The use of filler and three-layer tube rework reduces the cost price of the product by 15%.
3. The results show that although the mechanical strength of LDPE and HDPE is similar and that different proportions of these two polymer types do not cause a significant change in mechanical strength, given the low price of HDPE compared to LDPE, increasing the proportion of this material to 3.42 reduces the cost of the compound.
4. The use of a polyethylene blend with factory rework reduces the cost of the product by 35%–45% compared to Borostar. The properties of the finished tube are within the standard range.
5. All shock tubes have been successfully exploded without defects following tests for oil penetration resistance, bursting strength and heat aging.
6. The tensile strength of all shock tubes was within the standard range for high performance shock tubes (more than 170 N). The mechanical properties of the shock tubes did not differ significantly. However, despite the better mechanical properties of Borostar polyethylene in this study compared to the polyethylene alloy, shock tubes N5, N6 and N7 had better mechanical properties than shock tubes N1, N2, N3, N4 and N8. This discrepancy is probably due to the better adhesion of the Surlyn- and PEGMA-containing rework to the second layer of the shock tube.
7. The shock tubes N5, N6 and N7 showed higher linear thermal shrinkage than the shock tubes N1 to N4 at both 60 ℃ and 80 ℃ . This behavior can be attributed to the lower crystalline regions in the Surlyn and PEGMA structure.
8. In conclusion, the shock tubes N1, N2, N3, N4, N5, N6 and N7 have similar properties to the shock tube N8, so any of the blends P1, P2, P3, P4, P5, P6 and P7 can be a suitable alternative to Borostar.

## Data Availability

The original contributions presented in the study are included in the article/supplementary material, further inquiries can be directed to the corresponding author.
